# The Impact of Active Ingredients of Social Networks on the Trajectory of Cognitive Decline in Older Adults

**DOI:** 10.1093/geroni/igaf122.3000

**Published:** 2025-12-31

**Authors:** Jingchao Feng

**Affiliations:** Tsinghua University, Beijing, Beijing, China (People’s Republic)

## Abstract

Background Social networks, which are closely related to cognitive health and well-being in older adults, undergo significant changes as individuals enter old age. However, there is a lack of longitudinal studies examining the relationship between cognitive decline trajectories and the structural and qualitative aspects of social networks. Our study aims to explore this association and identify the influence of different social network dimensions across relationship types on cognitive trajectory development. Methods The Health and Retirement Study (HRS) is a nationally representative cohort study of older person in the United States. A psychosocial survey assessed the structural and qualitative aspects of social networks, including network size, close relationships, social support provided by the network, and social stress caused by the network. Cognitive function was measured on a 27-point scale every two years. This study employed group-based trajectory modeling to estimate cognitive decline trajectories and used multinomial logistic regression to analyze the association between trajectory categories and components of social networks. Results Among the 10,365 participants aged 50 and above at baseline, trajectory analysis identified three distinct longitudinal cognitive decline trajectories: Persistently low trajectory (n = 1,669, 16.1%), Persistently moderate trajectory (n = 5,058, 48.8%), Persistently high trajectory (n = 3,628, 35.1%). A larger social network and higher social support were protective factors associated with a slower cognitive decline and a higher trajectory. In contrast, social stress increased the risk of a rapid decline in cognitive function but also played a protective role in maintaining a higher cognitive trajectory.

